# Perspectives for Future Use of Cardiac Microtissues from Human Pluripotent Stem Cells

**DOI:** 10.1021/acsbiomaterials.1c01296

**Published:** 2022-03-22

**Authors:** Ulgu Arslan, Valeria V. Orlova, Christine L. Mummery

**Affiliations:** Department of Anatomy and Embryology, Leiden University Medical Centre, Einthovenweg 20, 2333ZC Leiden, The Netherlands

**Keywords:** pluripotent stem cells, human induced pluripotent stem cells, engineered heart tissue, cardiac microtissues

## Abstract

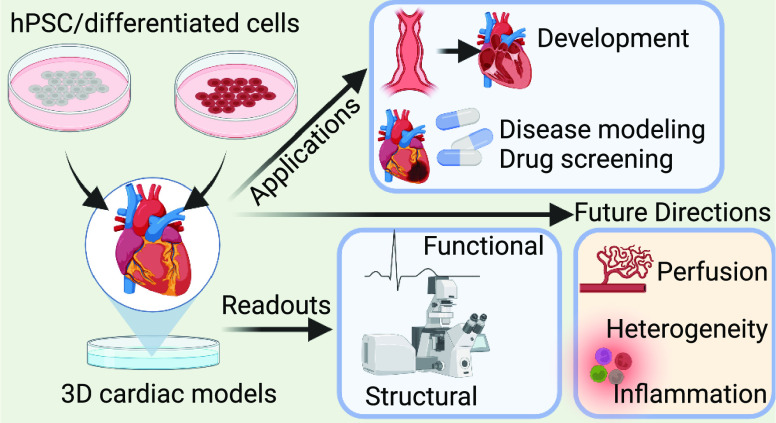

Cardiovascular disorders remain a critical health issue worldwide. While animals have been used extensively as experimental models to investigate heart disease mechanisms and develop drugs, their inherent drawbacks have shifted focus to more human-relevant alternatives. Human embryonic and induced pluripotent stem cells (hESCs and hiPSCs, collectively called hPSCs) have been identified as a source of different cardiac cells, but to date, they have rarely offered functional and structural maturity of the adult human heart. However, the combination of patient derived hPSCs with microphysiological tissue engineering approaches has presented new opportunities to study heart development and disease and identify drug targets. These models often closely mimic specific aspects of the native heart tissue including intercellular crosstalk and microenvironmental cues such that maturation occurs and relevant disease phenotypes are revealed. Most recently, organ-on-chip technology based on microfluidic devices has been combined with stem cell derived organoids and microtissues to create vascularized structures that can be subjected to fluidic flow and to which immune cells can be added to mimic inflammation of tissue postinjury. Similarly, the integration of nerve cells in these models can provide insight into how the cardiac nervous system affects heart pathology, for example, after myocardial infarction. Here, we consider these models and approaches in the context of cardiovascular disease together with their applications and readouts. We reflect on perspectives for their future implementation in understanding disease mechanisms and the drug discovery pipeline.

## Introduction

### Cell Sources for Human Cardiac Models

Animals like mice are tractable experimental models widely used in biomedical research to study the effects of disease and drugs. However, their distinct physiology, for example, in the contraction kinetics and ion channel regulation in the heart, limits the translation of outcomes to humans (reviewed in ref (1)). For this reason, there is increasing interest in developing more relevant models that recapitulate human (patho)physiology. Among these options are human pluripotent stem cells (hPSCs). hPSCs can divide indefinitely in culture and differentiate into all cells of the body including those of the heart. Since the first generation of human embryonic stem cells in 1998,^2^ research accelerated particularly over the past decade with the advent of somatic cell reprogramming that allows hPSCs to be derived from any healthy individual or patient as human induced pluripotent stem cells (hiPSCs).^3^ In many cases, defined media have been designed and become commercially available for robust culture and directed differentiation. To study development and disease in the human heart, it is now feasible to generate not only beating cardiomyocytes but also the specialized somatic cells of the heart like cardiac fibroblasts and cardiac endothelial cells.^4,5^ The heart additionally consists of smooth muscle cells, pericytes, immune cells, and (sympathetic/parasympathetic) neurons with different lineage origins (cardiac mesoderm, the proepicardium, and cardiac neural crest^6^), some of which can also be generated from hiPSCs. When combined in 2D or 3D formats, intercellular dialogue that is either paracrine or mediated by gap junction formation is initiated between these different cell types.^4^ This can result in maturation of the cardiomyocytes ,which like most hPSC-derivatives, are immature and fetal-like. As a result, exciting new opportunities are arising to capture features of healthy and diseased human tissues in cell culture based on stem cells. The challenge now is to transform these modalities into reproducible formats with tailored readouts and sensors to monitor cell physiology, preferably *in situ*.

### 2D and 3D Cardiac Models and Their Use

The simplest human stem cell cardiac models are 2D monolayer cultures of beating cardiomyocytes. These cells essentially have the full complement of ion channel genes and are widely used for screening potential cardiotoxicity of certain compounds on the heart.^7,8^ Certain drugs can cause sudden cardiac death by affecting ion channels (like the HERG channel), and individuals with mutations in ion channel genes can undergo fatal arryhthmia.^9^ Other compounds (such as some chemotherapeutics like doxorubicin) can cause heart failure.^10^

**Figure 1:**
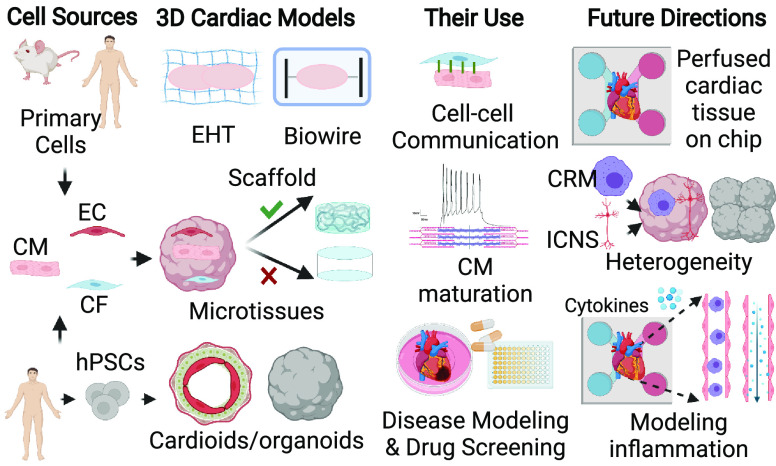
3D cardiac models and their use and future directions. hPSC, human pluripotent stem cell; CM, cardiomyocyte; EC, endothelial cell; CF, cardiac fibroblast; EHT, engineered heart tissue; CRM, cardiac resident macrophage; ICNS, intrinsic cardiac nervous system. This figure was created with BioRender.com.

The immaturity of hPSC-derived cardiomyocytes in 2D monolayer cultures is their drawback for modeling diseases or drug responses that affect the adult heart. In addition, where noncardiomyocyte cell types contribute to the disease phenotype, these simple monotypic 2D models might fall short. Here, more complex 3D models that incorporate stromal cells or mechanical and microenvironmental cues may be of value (Figure1). These include for example self-organized 3D cardiac microtissues, engineered heart tissues (EHTs), and “biowires”, which are assembled from predifferentiated hPSCs,^11,12^ or more recently, “cardioids”, derived by directed differentiation of hPSCs as cell aggregates^13^ and multicell type cardiac organoids.^14^ These models recapitulate several features of native heart tissue, such as 3D structure, contractile function, maturation state, and development. There are many variants of these basic models (recently reviewed in ref (15)), but they are essentially the groundwork for future implementation both in academia and in the drug development pipeline. The inclusion of perfusable vasculature in these models might increase their functionality even further (as discussed below), and the combination of (patient) hiPSC-derived cardiac models with organ-on-chip devices may enhance wider utility. Current organ-on-chip devices generally culture cells or tissues on permeable membranes or in 3D scaffold materials; they are beginning to be used to investigate cardiovascular disease or assess drug responses (reviewed in refs (16 and 17)).

### Readouts

Several studies have described assays of electrical activity (such as field potential duration measured by a microelectrode array^18^), calcium transients (measured semiautomatically using intracellular calcium-sensitive dyes^19^), or contraction (measured using software like MUSCLEMOTION to quantify movement on video images of contracting cardiomyocytes^20^). Simultaneous measurement of action potential, calcium transients, and contraction using dyes with appropriate sensitivities (“triple transient measurements”^21^) can predict drug responses in humans particularly accurately in both 2D and 3D models.

Aside from the functional analysis of simple and complex human cardiac tissues, the assessment of the structure can provide useful information on (changes in) cell behavior, distribution of specific cell types within the culture (for example, extent of self-organization), cellular crosstalk (for example, through gap junction formation), or cellular (ultra)structure (for example, sarcomere alignment and length). Widefield and confocal microscopy are widely used for this purpose but give low axial resolution and high photodamage so that imaging live cultures is particularly challenging. Light sheet microscopy (among multiple advanced imaging systems) is an emerging solution to this problem since it allows high speed and precise acquisition of images of (intra)cellular and tissue structures ranging from microns to millimeters in size.^22^

### Multicell Type Disease Models

As mentioned above, hPSCs can be derived from any individual so that when generated by reprogramming from a patient with an inherited cardiac condition (as hiPSC), it is possible to create multicell type cardiac microtissues of predifferentiated cardiomyocytes, cardiac fibroblasts, and cardiac endothelial cells from the mutant hiPSCs and/or their genetically corrected controls. Isogenic series of microtissues (or EHTs) can thus be generated in which just one of the (three) cell types carry the mutation. In this way, we have shown, for example, that for a genetic condition called arrhythmic cardiomyopathy (ACM) caused by a mutation in the desmosomal PKP2 gene, the simple introduction of mutant hiPSC–cardiac fibroblasts with healthy control cardiomyocytes and cardiac endothelial cells in a microtissue is sufficient to result in arrhythmias upon electrical pacing at rates >2 Hz.^4^ This is despite the cardiomyocytes being normal and indicates the cardiac fibroblasts can be the cellular “co-culprit” in this disease.

## Future Directions

From the above, we clearly see a wealth of *in vitro* models are now available to study the human heart that may well have benefits over mouse models because the cardiac cellular (patho)physiology is closer to that of patients. As a simple example, the human heart beats at ±60 times per minute and the mouse heart, at 500.^1^

How might we consider making these models have even broader applicability?

### Integration of Perfusable Microvascular Networks

Endothelium is essential in the heart as it provides a barrier for selective nutrient, oxygen, and drug delivery to heart cells and is involved in immune cell trafficking.^23^ Current 3D cardiac organoid and microtissue models often show evidence of organized microvasculature incorporation but usually depend solely on passive diffusion for external molecular and cellular access due to the lack of living or engineered microfluidic channel access. Transplantation of microtissues into live animals has been used as one way to promote vascular network invasion *in vivo* to form functional vasculature.^24,25^ Recently, however, advanced biofabrication techniques and microfluidic systems like organs-on-chip have been able to offer new opportunities to recapitulate this process *in vitro*. Vasculature can be engineered into tissue models using advanced bioassembly technologies, which include micropatterning by 3D bioprinting using synthetic or natural protein scaffolds or scaffold-free printing. Of note, scaffolds may limit cell-to-cell crosstalk within the tissue. Bioprinting usually defines the structural arrangement of cells such that there is coordinated organization of tissue components, and these tissue models become perfusable (reviewed in ref (26)). An alternative to bioprinting, which may be a closer mimic of transplantation *in vivo*, is to prevascularize microtissues. This can be done, for example, by coculturing (hiPSC-derived) vascular endothelial and smooth muscle cells with the microtissue components and then adding an external vascular network, which then self-organizes around and invades the microtissue. In a microfluidic organ-on-chip device, this would facilitate interconnection with pre-existing microvessels.^27^ This is called anastomoses: a biological process where branches of endothelium connect and form a continuous perfusable vascular network in the body. Intrinsic or extrinsic pro-angiogenic factors can direct endothelial cell growth for anastomoses in these *in vitro* models. When connected to microfluidic flow reservoirs, these systems could support perfusion of small molecules, growth factors, drugs, and/or immune cells from the external vascular network, for example, to allow better metabolic maturation of the tissue, modeling of inflammation, and drug screening.

### Integration of Other Cell Types to Achieve Heterogeneity in 3D Models

Even when cell types that enhance complexity and maturation are present in the microtissues, the addition of other cell types in different ways may further increase clinical relevance. Cardiac resident macrophages (CRMs), for example, regulate the electrical activity of cardiomyocytes by increasing their excitability and decreasing their refractory period through direct connection with cardiomyocytes via gap junctions that contain connexin 43.^28,29^ These maintain cardiac homeostasis by removing, among other things, dysfunctional mitochondria “ejected” by cardiomyocytes.^30^ There is compelling evidence for involvement of CRMs in collagen deposition to scar areas after damage and consequent fibrosis^31^ and cardiac regeneration,^32,33^ but their precise role still needs further investigation. In addition, the cardiac autonomic nervous system is a network of neurons that spreads from the brain to the heart and regulates the electrical and mechanical function of the heart.^34^ At the organ level, there is also a rich intrinsic cardiac nervous system (ICNS), which is mainly located in the cardiac ganglia.^34^ The ICNS is composed of sensory, motor, and interconnecting neurons that contribute to the heart rate, contractility, conduction, and blood flow.^35^ ICNS involvement in cardiac pathophysiology has been recognized previously;^36^ in some cases, a therapeutic option has been to sever these nerves, but the effects of nerve cells in 3D cardiac models *in vitro* are largely unexplored.^37,38^ This may help in understanding the dialogue between the nervous system in the heart and the cardiomyocytes.

Finally, tissue or spheroid/microtissue fusion or “stacking” of cardiomyocyte sheets can be used to create mechanical and cellular heterogeneity by altering cell density and other features such as size, shape, and conductivity.^39^ This may support elucidation of the role of tissue heterogeneity on structure and function in healthy and diseased states^40^ and offer new tools for regenerative medicine.^41^

### Modeling Inflammation in the Heart Following Damage or (Infectious) Disease

As a response to tissue injury, a complex cascade of events takes place at the injury site, which includes trafficking of immune cells, activation of fibroblasts, and synthesis of extracellular matrix components. These events suffice for tissue healing in cases of minor and nonrepetitive injury. However, where there is severe or repetitive injury, deregulation of the inflammatory response could lead to chronic inflammation and consequently endothelial dysfunction. This is a major contributor to heart failure. Besides genetic factors, cellular heterogeneity, and plasticity, cytokine release and dynamic cell–cell crosstalk, for example, between CRMs and T cells in infectious diseases or CRMs, cardiac fibroblasts, and endothelial cells in fibrosis, play important roles in mediating the inflammatory response.^42,43^ Due to the complex multicellular nature of pathophysiological and specifically human mechanisms underlying inflammation and the resulting disease, current *in vivo* models in mice may not be fit-for-purpose. There is therefore still an unmet need for *in vitro* models that can bridge the gap between *in vivo* studies and clinical trials. The advantages of microfluidic technologies and the incorporation of vasculature and relevant stromal and immune cells together with microenvironmental cues and perfusion may provide one with unique solutions to understand inflammation. Valuable information can thus be accrued on cell–cell interaction and cytokine mediation of the inflammatory response and potential therapeutic targets.

## Conclusions

We summarized here various 3D microphysiological systems that have been used to model human heart tissue, including cell sources, applications, and readouts. These models are already providing valuable insights into (patho)physiological mechanisms in cardiac development and disease. We also provide a perspective on how these models could be further improved to provide even better mimics of native human heart tissue: incorporating (vascular and immune cell) perfusion, creating greater cellular and mechanical heterogeneity by including noncardiomyocyte cell types, or using tissue fusion techniques. We expect these refinements to the models will enhance their utility for disease modeling and drug screening even further and perhaps support the discovery of treatments for presently incurable conditions of the heart such as chronic heart failure.
